# LICS: Locating Inter-Character Spaces for Multilingual Scene Text Detection

**DOI:** 10.3390/s26010197

**Published:** 2025-12-27

**Authors:** Po-Chyi Su, Meng-Chieh Lee, Yi-Ting Tung, Li-Zhu Chen, Chih-Hung Han, Tien-Ying Kuo

**Affiliations:** 1 Department of Computer Science and Information Engineering, National Central University, Taoyuan 320317, Taiwan; 2 Department of Electrical Engineering, National Taipei University of Technology, Taipei 106344, Taiwan

**Keywords:** deep learning, semantic segmentation, scene text detection, multilingual text localization, character recognition, weakly supervised learning, character gap

## Abstract

Scene text detection in multilingual environments poses significant challenges. Traditional detection methods often struggle with language-specific features and require extensive annotated training data for each language, making them less practical for multilingual contexts. The diversity of character shapes, sizes, and orientations in natural scenes, along with text deformation and partial occlusions, further complicates the task of detection. This paper introduces LICS (Locating Inter-Character Spaces), a method that detects inter-character gaps as language-agnostic structural cues, enabling more feasible multilingual text detection. A two-stage approach is employed: first, we train on synthetic data with precise character gap annotations, and then apply weakly supervised learning to real-world datasets with word-level labels. The weakly supervised learning framework eliminates the need for character-level annotations in target languages, substantially reducing the annotation burden while maintaining robust performance. Experimental results on the ICDAR and Total-Text benchmarks demonstrate the strong performance of LICS, particularly on Asian scripts. We also introduce CSVT (Character-Labeled Street View Text), a new scene-text dataset comprising approximately 20,000 carefully annotated streetscape images. A set of standardized labeling principles is established to ensure consistent annotation of text locations, content, and language types. CSVT is expected to facilitate more advanced research and development in multilingual scene-text analysis.

## 1. Introduction

**Figure 1:**
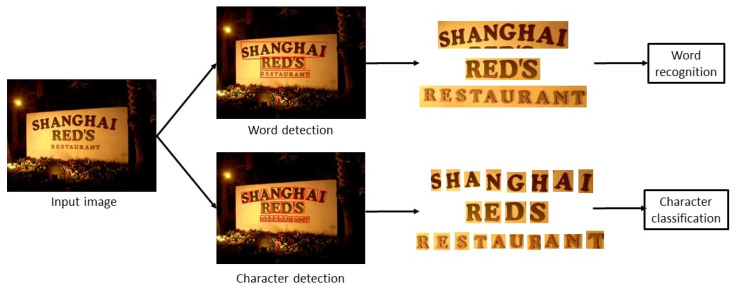
Common strategies of scene text detection and recognition.

Text plays a crucial role in communication and permeates every aspect of daily life. Natural-scene text refers to printed or written text found in the environment, such as road signs, shop signs, and notice boards, which provides valuable contextual information. Automating the extraction, recognition, and analysis of scene text from images can lead to the development of various convenient applications and services, including smart navigation, multilingual translation, and improved scene understanding. Text detection and recognition constitute the core pipeline for extracting information from scene images. As shown in Figure 1, character detection identifies individual characters, while word detection groups related characters into words. Accurately locating text allows subsequent recognition models to extract information more effectively, ultimately enhancing the efficiency and functionality of related applications.

**Figure 2:**
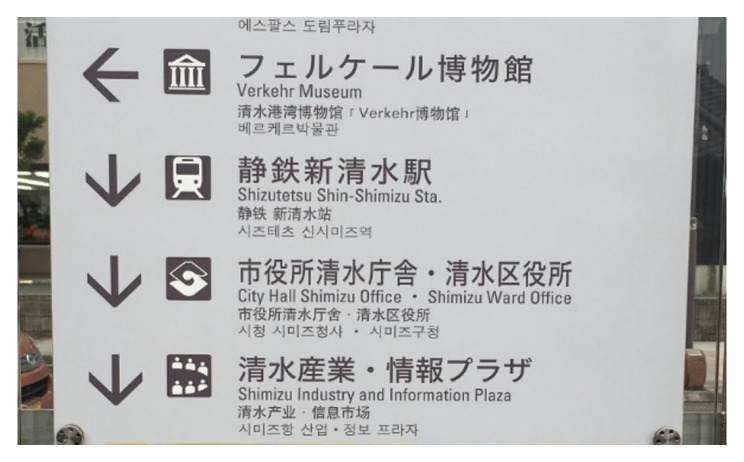
An example of a multilingual natural scene image.

Detecting text in natural scenes is a highly challenging task. Text in images can be affected by complex backgrounds, irregular orientations, varying sizes, blurring, and text-like patterns, all of which complicate accurate detection. Multilingual text frequently appears in such environments, as shown in Figure 2. Existing methods are often trained for specific languages, limiting their adaptability to scenes containing multiple languages. Moreover, these methods typically require extensive data collection and annotation for each target language. This research aims to develop text detection methods that can effectively handle multilingual scenarios, improving natural-scene text parsing while reducing language-specific limitations.

Most scene text datasets rely on word-level annotations, which simplify the labeling process. Words are typically enclosed in bounding boxes defined by four points, providing clearer contextual and semantic information. However, word-level annotations have certain drawbacks: segmentation can vary depending on annotators’ interpretations, and models trained on word-level annotations often require larger datasets, increasing annotation and training costs. In contrast, character-level annotations are more intuitive and consistent. Character detection can independently locate characters across different languages, facilitating subsequent recognition while reducing the influence of complex backgrounds. This enables the development of lightweight models capable of achieving accurate text recognition using character-level annotations. Character-level labeling is also better suited for multi-oriented text in natural scenes and reduces ambiguity during annotation. The main drawback is that annotating individual characters is time-consuming, as each word may contain multiple characters, which is why most datasets still rely on word-level annotations to reduce workload.

It is important to note that directly detecting characters can make model training more language-specific. For example, a model trained on English alphanumeric characters may struggle with Chinese characters due to differences in shape, structure, and arrangement, which can reduce the model’s generalization across languages. The choice between word-level and character-level detection also affects subsequent recognition. Word recognition often requires preprocessing, such as adjusting the word orientation to horizontal for proper recognition. In contrast, character recognition is less sensitive to orientation, as individual characters can be effectively localized with four-point boxes regardless of their positioning. Additionally, character boxes typically include less background, allowing for more direct and reliable recognition.

This research proposes a method for character localization in natural scenes based on detecting inter-character gaps, referred to as LICS (Locating Inter-Character Spaces). LICS focuses on the spaces between characters, which exist in both printed and handwritten text. Since inter-character gaps are universal features, they are less dependent on specific language glyphs or structures. This allows gap detection to transcend language limitations, enhancing the generalizability of character detection models across various scripts, including English alphanumeric, Chinese, Japanese, and Korean characters. LICS leverages this property to perform character localization in multilingual natural scene images using a training dataset containing only synthetic annotations of alphanumeric characters and word-level annotations for other target languages. Specifically, detecting inter-character gaps serves as a preprocessing step for multilingual character detection. Combined with weakly supervised learning, this approach addresses the lack of character-level annotations in real multilingual image datasets. We evaluated LICS on multilingual datasets to validate its effectiveness across diverse real-world scenarios.

**Figure 3:**
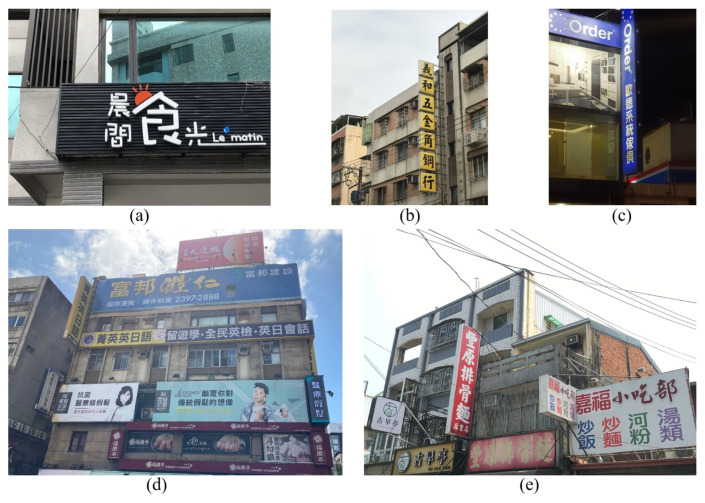
Some examples of the CSVT dataset, including simple street view (**a**–**c**) and complex street view (**d**,**e**).

In addition, we introduce a new dataset for scene text analysis, Character-Labeled Street View Text (CSVT), containing approximately 20,000 carefully annotated images. To ensure consistent annotation of text locations, contents, and language types (Chinese and English), a standardized set of labeling principles was established. All images in CSVT were collected using mobile phones of various brands in cities across northern Taiwan. The captured scenes exhibit substantial variation in signboard heights, shapes, background colors, patterns, fonts, sizes, orientations, and arrangements. Figure 3 presents several typical examples from CSVT, illustrating the dataset’s diversity and complexity.

LICS can thus enable a wide range of real-world scene-text applications because it treats the gaps between characters as a language-agnostic cue, reducing the need for expensive character-level labeling when moving across scripts. That makes it especially useful for multilingual OCR pipelines in the wild, such as reading street signs, storefronts, public notices, and transportation wayfinding. It also supports capabilities such as smart navigation aids, instant translation, and richer scene understanding in consumer apps and augmented-reality overlays. In safety- and mobility-oriented systems, such as driver assistance and robotics, more reliable multilingual text localization can improve map awareness and contextual decision-making from signage. LICS can also enhance downstream recognition by producing cleaner character segmentation, which is valuable for indexing and searching large image collections (such as street-view archives, retail audits, and compliance inspections) and for building better multilingual datasets and benchmarks, particularly through resources like the CSVT dataset introduced alongside the method.

The remainder of this paper is organized as follows. Section 2 reviews related work in the field of text detection. Section 3 presents the proposed inter-character gap detection method and the weakly supervised learning approach. Section 4 introduces our scene text dataset. Section 5 reports the experimental results evaluating the proposed scheme. Finally, Section 6 concludes the paper and discusses potential directions for future work.

## 2. Related Work

Early scene-text detection methods primarily relied on manual image-processing techniques, including handcrafted feature extraction and heuristic rules. Common approaches involved color conversion, edge detection, thresholding, and Hough transforms. While effective for constrained scenarios, these methods lacked the flexibility to handle the diversity and complexity of real-world scenes. Recent advances have shifted toward deep learning-based approaches, which allow models to automatically learn rich visual features, significantly improving text-detection performance. Some representative works are reviewed below.

### 2.1. Object Detection Methods vs. Semantic Segmentation Methods

Deep learning-based scene text detection methods can be broadly categorized into object-detection-based and semantic-segmentation-based approaches. Object-detection-based methods treat text as visual objects and localize text regions via bounding boxes. Early models such as Faster R-CNN [1], SSD [2], and YOLO [3,4] perform well on well-structured text but struggle with irregular, overlapping, or densely packed text. Specialized methods for scene text detection include EAST [5], which predicts text boxes using anchors and merges multi-resolution features with Non-Maximum Suppression (NMS) [6]; CTPN [7], which combines VGG16 [8] and LSTM [9] to capture context and generate multi-scale text proposals; and PAN++ [10], which enhances features with pyramid modules and redefines text as a central region surrounded by peripheral pixels for detecting arbitrarily oriented text. MOST [11] further refines bounding boxes using Text Feature Alignment (TFAM) and Position-Aware NMS (PA-NMS). Transformer-based [12] object-detection methods have recently advanced the flexibility of text localization. DPText-DETR [13] replaces bounding-box predictions with Explicit Point Query Modeling (EPQM), iteratively refining point-based queries, while its Enhanced Factorized Self-Attention (EFSA) module uses ring convolutions to model polygonal text regions, enabling accurate detection of curved and arbitrarily oriented text.

Semantic-segmentation-based methods offer pixel-level classification, providing a finer delineation of text regions. Foundational models include FCN [14] and U-Net [15]. DeepLab [16] uses dilated convolutions and Atrous Spatial Pyramid Pooling (ASPP) to capture multi-scale context, while PSPNet [17] employs Pyramid Pooling for global context aggregation. Mask R-CNN [18] extends Faster R-CNN with a mask prediction branch for pixel-level segmentation, and DBNet [19] introduces Differentiable Binarization to convert text scores into binary masks. The SIR model [20] integrates Global-Dense Semantic Contrast (GDSC) and combines Top-Down Modeling with Bottom-Up features for improved detection. Transformer-based segmentation approaches such as SwinTextSpotter [21] and SRFormer [22] enhance text detection by integrating global context. SwinTextSpotter treats detection as a set-prediction problem, combining detection and recognition to suppress background noise. SRFormer utilizes dual decoders, one for semantic segmentation and one for object detection, leveraging both high-level and pixel-level features with a Transformer encoder for precise localization.

### 2.2. Character-Level Processing

Most text detection methods operate at the word level, but CRAFT [23] focuses on character-level detection. It uses an FCN with skip connections to combine low-level image details and high-level semantic features, producing character region and affinity maps. Weakly supervised learning enables training without character-level annotations; however, its loss function considers only the number of detected characters, which may lead to misalignment between predicted and actual positions. Despite this limitation, CRAFT’s character-based approach significantly improves text detection accuracy and has influenced subsequent research in the field. CharNet [24] is a one-stage, character-level network that jointly performs text detection and recognition. It has two main components: the Text Detection Branch, which identifies text regions and adapts to multi-oriented or curved text (e.g., Char-Net [25] uses modified EAST/TextField [26] with directional fields), and the Character Branch, which employs synthetic data and iterative detection for weakly supervised training, including sub-branches for word binarization, character detection, and recognition. Iterative detection improves generalization and performance. I2C2W [27] divides the task into Image-to-Character (I2C) and Character-to-Word (C2W) stages. I2C predicts positional character embeddings, while C2W groups them into words. Transformers’ self-attention captures global character relationships, enabling accurate localization. C2W uses only word-level annotations for character positions. A Connectionist Temporal Classification (CTC) decoder [28] then generates the recognized words.

### 2.3. Weakly Supervised Learning

Weakly supervised learning and fully supervised learning primarily differ in the quantity and quality of labeled data. In supervised learning, all training samples are fully labeled, meaning that each input is paired with its corresponding ground truth. While this approach can achieve high accuracy, it requires substantial time and resources to generate these labels, particularly in domains that demand specialized expertise. In contrast, weakly supervised learning leverages partially labeled or incompletely labeled data, allowing the model to benefit from unlabeled samples when labeled data are scarce. Typically, a small set of labeled data is first used to train an initial model, which then generates pseudo-labels by making predictions on unlabeled data. These pseudo-labeled samples are combined with the original labeled data to retrain the model. Through multiple iterations, the model gradually refines its pseudo-labels, improving overall performance. The key advantage of pseudo-labeling is that it reduces reliance on extensive manual labeling. However, its effectiveness depends heavily on the accuracy of the initial model; poor initial predictions can propagate errors during subsequent training. Additionally, because pseudo-labels may deviate from true labels, robust evaluation mechanisms and strategies for updating pseudo-labels are critical to ensure reliable and effective model learning.

The weakly supervised learning approach used by CRAFT [23] involves training an initial model on a combination of real datasets with word-level annotations and synthetic character datasets. The model first utilizes the annotated word bounding boxes to identify text regions within the images. After the initial detection of character positions, the model generates bounding boxes for each character, which serve as pseudo-labels. A Character Region Score Map, combined with Connected Component Labeling, is employed to extract potential text regions. These regions are subsequently segmented into individual character areas. The predicted number of characters in each region is then compared with the ground-truth character count from the word-level annotations, and a confidence value, *C*, is calculated using (1).

(1) $$C=1-\frac{|N_{pred}-N_{true}|}{N_{true}},$$

where $N_{pred}$ denotes the number of characters predicted by the model, and $N_{true}$ represents the actual character count. This adjustment helps reduce the negative impact of discrepancies in character counts on model training. Similarly, CharNet [24] and Char-Net [25] leveraged large-scale synthetic datasets to train their initial models. However, due to differences between synthetic and real-world scene images, an iterative process is required, involving comparisons of both the number of characters and the recognized text content.

### 2.4. Related Datasets

**Figure 4:**
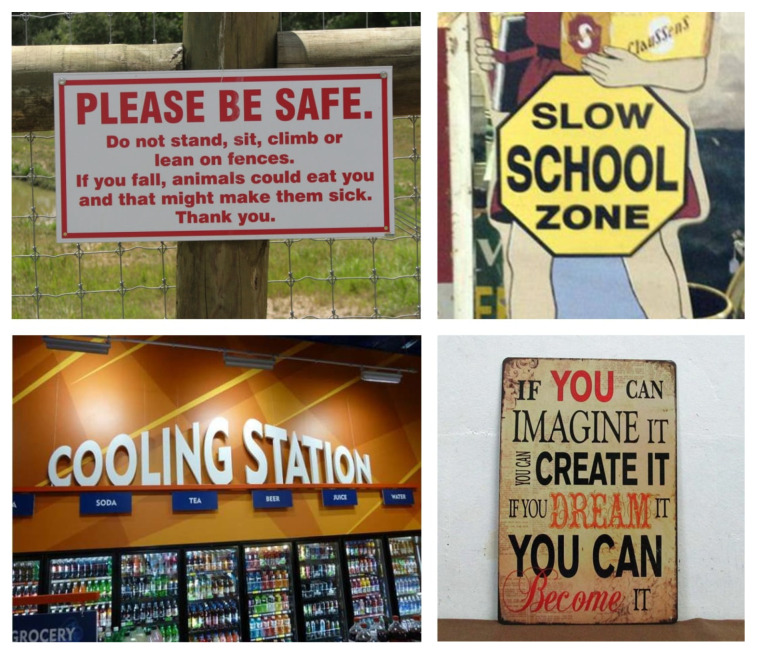
Examples of images in the ICDAR datasets.

Several datasets are dedicated to natural-scene text localization, with the ICDAR (International Conference on Document Analysis and Recognition) series being the most widely used. ICDAR, a prestigious international conference, has organized competitions on text localization and released datasets such as ICDAR2013 [29], ICDAR2015 [30], ICDAR2017 [31], and ICDAR2019 [32]. These datasets cover diverse application scenarios, featuring text in various styles, fonts, and sizes from books, documents, street scenes, and other natural environments, as illustrated in Figure 4. Each image is annotated with bounding boxes around text regions, along with the corresponding text content, including English, numerical digits, and languages such as Chinese, Japanese, and Korean.

**Figure 5:**
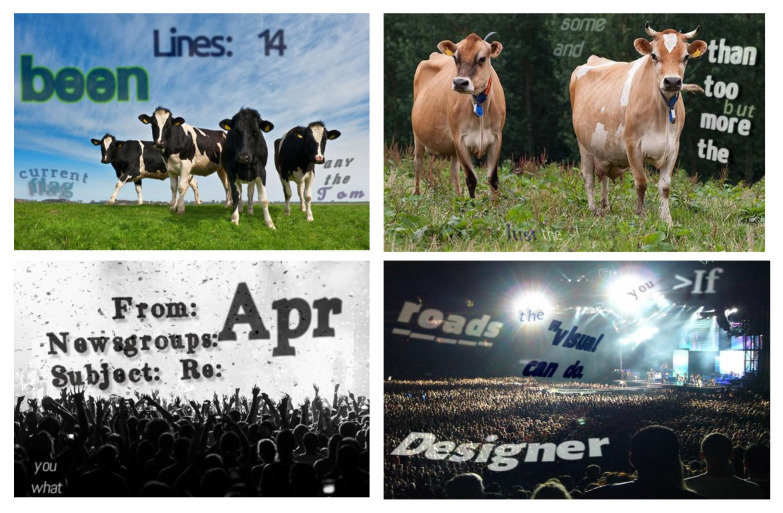
Examples of images in the SynthText dataset.

As previously noted, annotating text in real images is costly and often yields limited data. To address this, many synthetic datasets have been created, such as SynthText [33], which contains approximately 800,000 synthetic images simulating around eight million words in natural scenes with diverse text styles and sizes, as shown in Figure 5. SynthText provides detailed annotations at the string, word, and character levels. The abundance of synthetic images significantly expands the available training data, which is crucial for effectively training deep models. Additionally, synthetic datasets allow precise control over annotations, making them particularly suitable for supervised training and highly beneficial for the initial stage of weakly supervised learning, where an initial model is developed.

## 3. The Proposed Method

**Figure 6:**
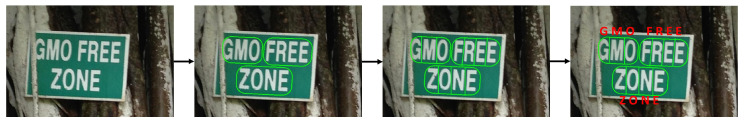
The methodology used in LICS for scene text analysis is illustrated from left to right, showing the input image, word detection, character localization, and character recognition.

The methodology of the proposed scheme, LICS, is illustrated in Figure 6. For each input image, we first apply segmentation-based word detection to identify text regions, which are subsequently divided into individual character regions for recognition. Segmentation-based detection is chosen for its flexibility in accurately capturing text areas. Word and character detection can be performed either sequentially or in parallel. In the sequential approach, words are detected first, followed by segmentation into individual characters. In the parallel approach, words and characters are detected simultaneously, with character locations determined jointly. While sequential processing may require more time, it remains practical for real-world applications, whereas parallel detection offers a simpler implementation.

**Figure 7:**
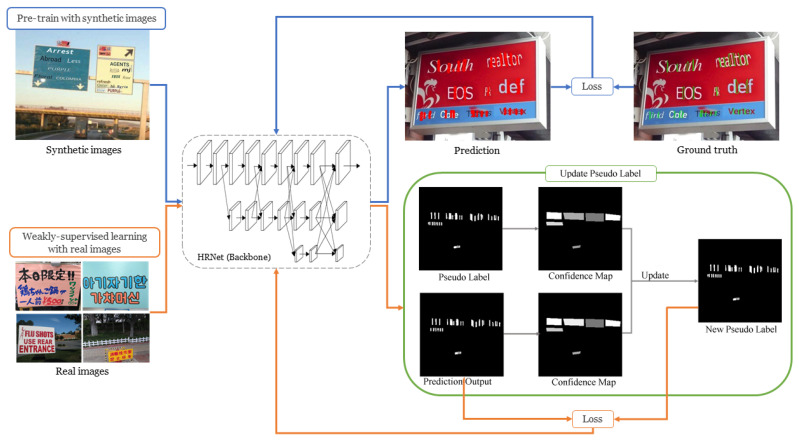
The model training in LICS. The blue arrows illustrate the initial model training process, whereas the orange arrows denote the weakly supervised learning procedure.

Accurate character detection relies on training an effective deep-learning model. The architecture used in our model training is illustrated in Figure 7. We adopt a semantic segmentation approach for inter-character gap detection, assigning each pixel in the image to a specific category to generate a pixel-level labeled map. Since our primary detection targets are the gaps between characters in multilingual scenarios, which are essential for locating character centers, we employ HRNet [34] as the backbone. HRNet is particularly well-suited for fine-grained semantic segmentation and precise localization because it maintains high-resolution representations through multiple parallel branches that process feature maps at different resolutions. The resulting multi-scale feature fusion improves the network’s ability to handle complex backgrounds and subtle variations in character gaps.

We use a synthetic dataset that closely mimics real-world natural scenes to automatically generate precise inter-character gap labels. For a text line composed of characters $C=\{c_{1},c_{2},\ldots ,c_{n}\}$, the inter-character gaps are defined as the regions separating each adjacent pair $\left(c_{i},c_{i+1}\right)$. Since synthetic images provide ground-truth character boundaries, the exact positions of these gaps can be computed automatically, without manual annotation. To improve performance on real-world images, we combine this synthetic data with real-world datasets containing only word-level annotations through a weakly supervised learning approach. This strategy allows the network to be fine-tuned and better adapt to detecting inter-character gaps in natural scene images. The detector is trained to output a gap-probability heatmap, where each pixel indicates the likelihood of belonging to an inter-character boundary. Training is guided by a mask derived from the synthetic gap annotations. After inference, the continuous heatmap is converted into discrete gap candidates using thresholding followed by non-maximum suppression.

Next, we describe the key components of LICS.

### 3.1. Synthetic Dataset and Annotation

**Figure 8:**
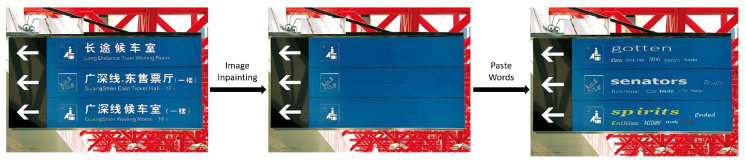
Generating synthetic images with gap bounding boxes: from left to right, an original image from an existing dataset, the inpainted image, and the resulting synthetic image.

We generate synthetic datasets that closely mimic real-world natural scenes while incorporating inter-character gap annotations, as illustrated in Figure 8. First, we compile a reference dictionary by downloading commonly used English words from online sources. Next, we utilize images from three datasets, including ICDAR2013, ICDAR2019-Art [35], and CSVT, to create synthetic images. To ensure that the synthetic images resemble natural scenes, we first remove the original words in images containing word-level annotations. This is achieved using the image inpainting functionality of PyPatchMatch [36], which fills in missing regions or removes unwanted objects. After erasing the original words, we overlay the images with randomly selected words. To simulate the varied appearances of words in real-world scenarios, we apply affine transformations and image warping using “createThinPlateSplineShapeTransformer” in OpenCV (ver. 4.12.0). These transformations allow the words to be positioned more realistically and with greater diversity, reducing the gap between synthetic and real data. This approach not only enhances the diversity and detection accuracy during network training but also enables the generation of realistic synthetic data for any language.

**Figure 9:**
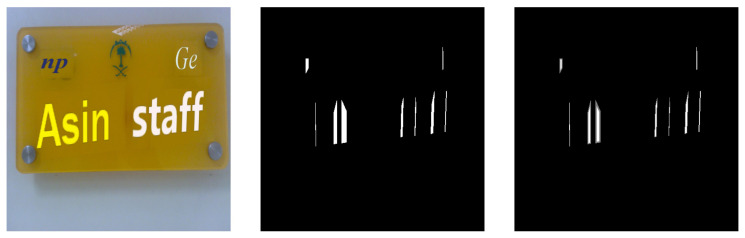
Data labeling: the original image is shown on the left, the binary classification labels in the middle, and the ternary classification labels on the right.

In the proposed scheme, character gaps are labeled as the detection targets, while all other areas are treated as background. In most text detection tasks using semantic segmentation, the standard approach is to label text regions and background as two separate classes, resulting in a binary classification network. However, our experiments showed that binary classification performed poorly for this scheme, likely due to the small size of the detection targets. To address this, we adopted the approach from CharSpotter [37] and extended binary classification to ternary classification. This method distinguishes between the gap center, the gap edge, and the background, as illustrated in Figure 9. To generate the three classes, we apply a Distance Transform by calculating the distance between each pixel (x,y) inside the gap box and its nearest background pixel (i,j) as $D\left(x,y\right)=\sqrt{{\left(x-i\right)}^{2}+{\left(y-j\right)}^{2}}$. The distances are then normalized to obtain H(x,y), where pixels closer to the center of the character gap approach 1, and those nearer to the background approach 0. Using thresholding, pixels with $H\left(x,y\right)>T_{1}\left(=0.6\right)$ are labeled as “gap centers,” pixels with $H\left(x,y\right)<T_{2}\left(=0.01\right)$ are labeled as “background,” and the remaining pixels are labeled as “gap edges.” The values are chosen empirically based on qualitative inspection to ensure stable separation between gap centers, edges, and background.

### 3.2. Network Architecture

**Figure 10:**
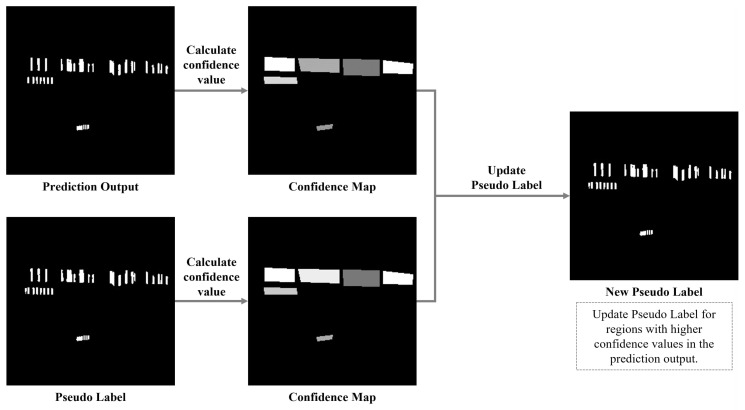
We evaluate the results using the pseudo-labels to generate confidence maps, and retain the regions with higher confidence as the updated pseudo-labels.

The network architecture in this study, illustrated in Figure 7, is organized into two stages. The first stage, indicated by the blue arrows, trains an initial model using a synthetic dataset. The second stage, represented by both the blue and orange arrows, fine-tunes the initial model with a real dataset, employing pseudo-labeling to adapt to the complexities of natural scene images and improve feature learning. Given that the pseudo-labels in the real images may have significant positional errors, we adjust the proportion of real images in each training batch to mitigate the negative impact of these errors and maintain model stability. When synthetic data is used as input, the model’s predictions are directly compared with the gap annotations to compute the loss. For real images, which contain only word-level annotations, a pseudo-label update mechanism is employed. More specifically, we evaluate the model’s predictions in conjunction with the gap detection results from the pseudo-labels. This process produces two “Confidence Maps,” which indicate the model’s confidence in each predicted target, with values ranging from 0 (low confidence) to 1 (high confidence). Regions with higher confidence values from these maps are retained as the updated pseudo-labels, as illustrated in Figure 10. The confidence value for each word box is visualized using a grayscale, where higher confidence is represented by lighter colors (closer to white) and lower confidence by darker colors (closer to black). For instance, in the second word box in Figure 10, the model’s prediction exhibits higher confidence than the corresponding pseudo-label, leading us to retain the model’s prediction for that box. After this update, each word box in the new pseudo-labels displays higher confidence levels, which are then used in the next round of comparisons.

### 3.3. Pseudo-Label Update

**Figure 11:**
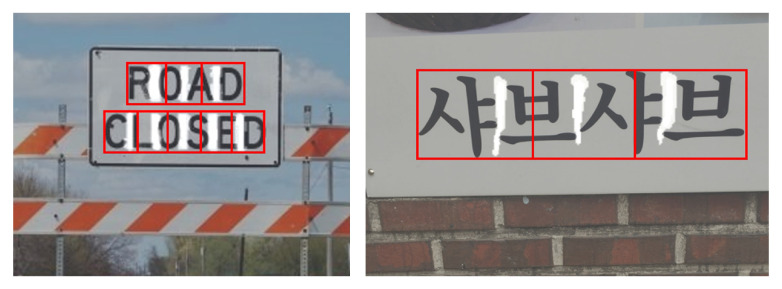
We divide the word box according to the number of gaps, expecting each region to contain a single connected component corresponding to a white character gap.

We update the pseudo-labels by evaluating the gap detection results based on both the number and positions of the gaps, which allows us to calculate a confidence value for each word box. Since most real-world scene image datasets provide only word box annotations and the corresponding text content, the evaluation relies solely on these available labels. Specifically, for each word box, we first check whether the number of detected gaps is correct. The expected number of gaps is N−1, where *N* is the number of characters in the word. Accordingly, each word box is divided into N−1 regions, with the expectation that each region contains one gap, as illustrated in Figure 11. For example, a word box containing four characters would be divided into three regions, each expected to contain a single gap. However, considering only the number of gaps is insufficient, as the model might predict the correct number of gaps while misaligning their positions with the actual character gaps. To address this, we also evaluate the likely locations of gaps within each word box. Since character size and font type can influence the positions and widths of gaps, we analyze multiple word boxes and adopt a reference proportion (approximately one-third of the potential character width within each box) to guide the expected gap positions. This approach helps refine pseudo-labels and improves the model’s accuracy in localizing character gaps.

**Figure 12:**
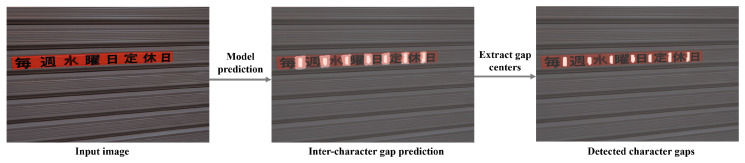
An example of character gap detection.

We expect each word box to contain exactly one gap between consecutive characters, resulting in a total of N−1 gaps for a word with *N* characters. If a region contains fewer or more than one gap, the model considers it a prediction error. In addition to the number of gaps, the accuracy of the predicted gap positions must also be evaluated. To do this, we extract the pixels corresponding to the gap centers from the model’s three-class output, as illustrated in Figure 12. After identifying connected components and confirming their spatial distributions, the word box is divided into 2N−1 regions: *N* character regions and N−1 gap regions, with the gap width approximately one-third of the character width. Ideally, the connected components of detected gap pixels should appear only in the gap regions and not within the character regions. The confidence value for each word box, $W_{\mathrm{Conf}}$, is calculated as:

(2) $$W_{\mathrm{Conf}}=\frac{1}{N}\times \sum_{i=1}^{N-1}\left(\frac{1}{C_{2i-1}}\right)-\lambda \times \frac{1}{N}\times \sum_{i=1}^{N}\left(\frac{1}{C_{2i-2}}\right),$$

where *N* is the number of characters in the word, and N−1 is the total number of gaps. The index *i* denotes the ith region, while $C_{2i-1}$ and $C_{2i-2}$ represent the number of connected components in the gap and character regions, respectively. Regions with zero connected components are disregarded. The parameter λ is a penalty coefficient. A confidence value closer to 1 indicates that the model’s predictions align more closely with the ideal gap positions. Calculating $W_{\mathrm{Conf}}$ for each word box in real images enables iterative refinement of the pseudo-labels.

### 3.4. Simulated Gaps

**Figure 13:**
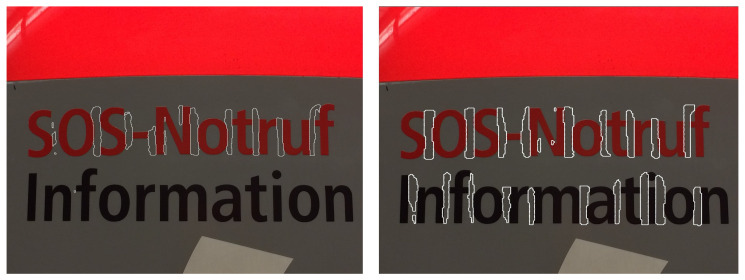
The left image shows the detection results at epoch 83 without simulated gaps, while the right image shows the results at the same epoch with simulated gaps applied.

Due to the lack of character gap annotations in real natural scene images, initial pseudo-labels for these images are generated using a model trained on synthetic data. However, these annotations may not fully capture the complexities of real-world backgrounds, leading to discrepancies. To improve accuracy, a word mask is created using the coordinates of the word annotations, filtering out pseudo-labels that fall outside the word boundaries. Gap localization is further refined using confidence maps, updating pseudo-labels to better align with real-world conditions. To facilitate rapid gap localization in real images, simulated gap positions are introduced to assist the model during training. These simulations are used solely for loss calculation and do not serve as actual annotations. As previously mentioned, each word box is assigned a confidence value reflecting the model’s certainty, with values close to 1 indicating strong alignment with true gap locations. A threshold of 0.5 is applied; if a word box has a confidence value below this threshold, simulated gaps are generated within the box. Gap simulation leverages the word content along with common fonts and font sizes matched to the word box dimensions. This aids in loss calculation and accelerates the model’s learning of gap localization. Figure 13 illustrates an example, showing that applying simulated gaps significantly improves results after the same number of epochs. It is important to note that using simulated gaps as actual pseudo-labels could artificially inflate confidence scores, potentially disrupting the iterative update of pseudo-label positions.

### 3.5. Loss Function

The loss function *L* is defined as the product of the classification loss $L_{c}$ and the confidence value $W_{\mathrm{Conf}}$ derived from the pseudo labels:

(3) $$L=L_{c}\times W_{\mathrm{Conf}},$$

where the confidence value $W_{\mathrm{Conf}}$ reflects how well the pseudo labels generated by the model align with the ideal gap positions in real images. A higher $W_{\mathrm{Conf}}$ indicates closer agreement with the ground truth. During the weakly supervised learning phase, which incorporates both synthetic and real images, synthetic samples are assigned $W_{\mathrm{Conf}}=1$ because their annotations provide precise gap locations.

To compute the classification loss $L_{c}$, we use the standard cross-entropy formulation:

(4) $$L_{c}=-\frac{1}{P}\left(W_{c}\times g_{i}^{c}\times log\left(s_{i}^{c}\right)\right),$$

where *P* is the total number of pixels in the image, *c* denotes the class index, $g_{i}^{c}=1$ if pixel *i* belongs to class *c* (and 0 otherwise), and $s_{i}^{c}$ is the predicted probability that pixel *i* belongs to class *c*. The loss is averaged over all pixels. Lower cross-entropy values indicate predictions that are more consistent with the true labels.

Pixels are categorized into three classes: gap centers, gap edges, and background. Since gaps occupy a relatively small area compared to the background, the dataset exhibits significant class imbalance. Without adjustment, the model may become biased toward predicting background pixels. To address this, we assign distinct class weights $W_{c}$, giving higher weights to gap centers and gap edges. In our scheme, the weights are set as $W_{\mathrm{background}}=1$, $W_{\mathrm{gap}\;\mathrm{edge}}=10$, and $W_{\mathrm{gap}\;\mathrm{center}}=15$. These weights encourage the network to focus more on gap-related classes, thereby improving detection accuracy.

### 3.6. Post-Processing

**Figure 14:**
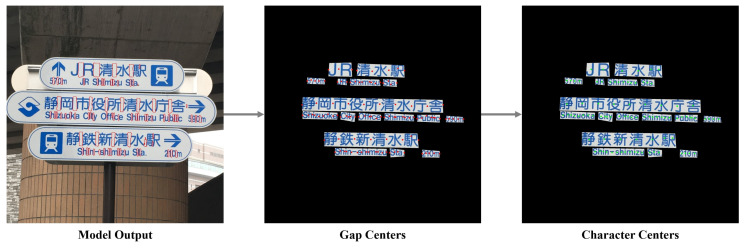
Utilizing the gap centers to locate the character centers.

**Figure 15:**
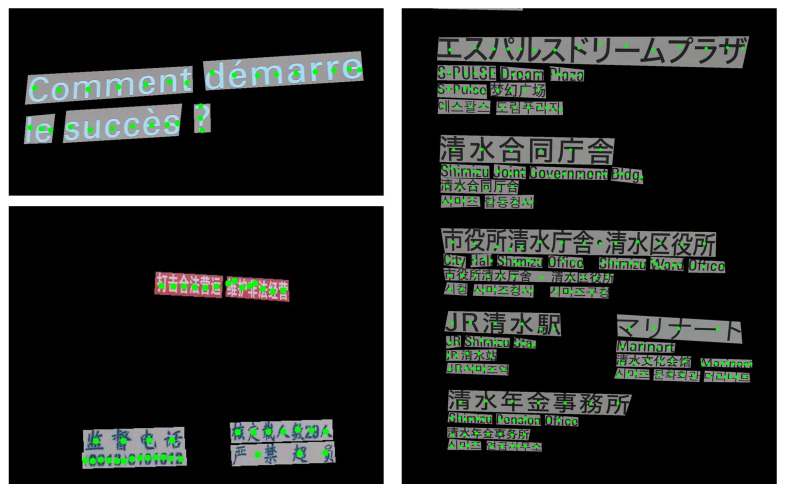
Utilizing the gap center to locate the character centers.

Since the model primarily detects gaps rather than character centers, we combine the detected gaps with word bounding boxes to accurately determine character locations. First, the gap center regions are extracted from the model’s detection results, and the edge coordinates of each gap region are used to precisely locate the gap center. The word bounding box then defines the overall boundaries of the word. To enhance accuracy, gap centers that may result from noise or incorrect detections are filtered out, retaining only those closest to the expected positions. This yields more precise character center locations, as illustrated in Figure 14. Once the character centers are established, as shown in Figure 15, they serve as the basis for subsequent single-character segmentation.

**Figure 16:**
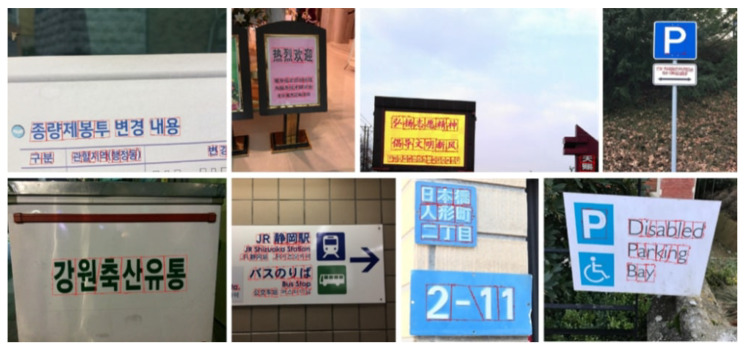
The results of character segmentation.

After determining the character centers based on the gap centers, we apply the Nearest Neighbor Algorithm to assign pixels from the text regions to their closest character centers. Specifically, the Euclidean distance between each pixel and all detected character centers is calculated, and each pixel is assigned to the nearest center. This procedure produces precise segmentation and coordinate information for each character region, as illustrated in Figure 16. The process begins by extracting the text regions from the original image using the word bounding boxes. Distances are then computed only for pixels within these regions relative to the nearest character centers. By restricting processing to relevant text areas, this approach avoids analyzing the entire image, thereby improving computational efficiency.

## 4. A Dataset for Scene Text Analysis, CSVT

### Types of Images

The images in the Character-Labeled Street View Text (CSVT) dataset can be broadly categorized into two types: simple and complex street-view scenes, as illustrated in Figure 3. Simple street-view images typically feature close-up views of store signs, with text lines clearly visible and minimally occluded. The text is generally oriented in either landscape or portrait directions, and the overall scene structure is relatively straightforward. In contrast, complex street-view images contain multiple lines of text with varying orientations and may include partial occlusions from objects such as wires or signboards, making text recognition more challenging. CSVT images were captured using mobile phones from various brands across different locations in northern Taiwan. As a result, the images vary in resolution, aspect ratio, and other imaging settings. To standardize the dataset for analysis, all images are resized so that the shorter side is fixed at 1024 pixels, with the longer side scaled proportionally to preserve the aspect ratio. The dataset provides comprehensive annotations for all text lines and characters within each image. Each text element is recorded using UTF-8 encoding and assigned a category ID based on the language. CSVT supports multilingual text recognition, particularly for Chinese and English. Each text location is annotated as a four-point polygon arranged in clockwise order, starting from the top-left corner. The resulting ground-truth (GT) annotations are provided in a JSON file.

The annotations in the CSVT dataset are organized into several language categories, as listed in Table 1, with a primary focus on Chinese text and English alphanumeric characters. Categories 0 and 1 correspond to Chinese strings and Chinese characters, respectively, which are the most frequently occurring elements in the dataset. Category 2 contains English alphanumeric strings, while Category 3 includes mixed strings containing both Chinese and English alphanumerics. Category 4 is reserved for single Chinese characters. This distinction between Chinese strings, individual Chinese characters, and single-character cases facilitates recognition across diverse scenarios.

Categories 5 and 255 may include blurred or otherwise difficult-to-read text. The key difference is that text in Category 5 remains partially discernible, while text in Category 255 is largely unrecognizable, though its presence can still be perceived. Consequently, Category 5 samples can be used to train sensitive text-detection models but are excluded from text-recognition tasks. Additionally, text in other languages (e.g., Japanese, Korean, Thai), completely mirrored or reflected text, and very small text are grouped into Category 5, as annotators may lack familiarity with these languages or find the text too difficult to interpret. Category 255 is fully excluded from both text detection and recognition, and evaluation ignores predictions on these instances. Text is assigned to Category 255 under any of the following conditions: (1) more than 20% of the text is occluded; (2) the text content is ambiguous or controversial; (3) mirrored or reflected text is incomplete; or (4) multi-line text is blurred beyond recognition.

Vehicle license plates (Category 6) and faces (Category 7) are annotated primarily for privacy purposes. Portions of images containing these elements are blurred to prevent privacy violations. For content annotation, characters within strings are entered from left to right, consistent with the reading order of most horizontal text lines. English letters, numbers, and punctuation are recorded in half-width form, and spaces between words are ignored. For strings classified as Category 5 or 255, the content is represented as “###” to distinguish it from the literal “#” character that may appear in text. If the bounding boxes of multiple strings overlap, the content of the primary string is retained as the annotation.

**Figure 17:**
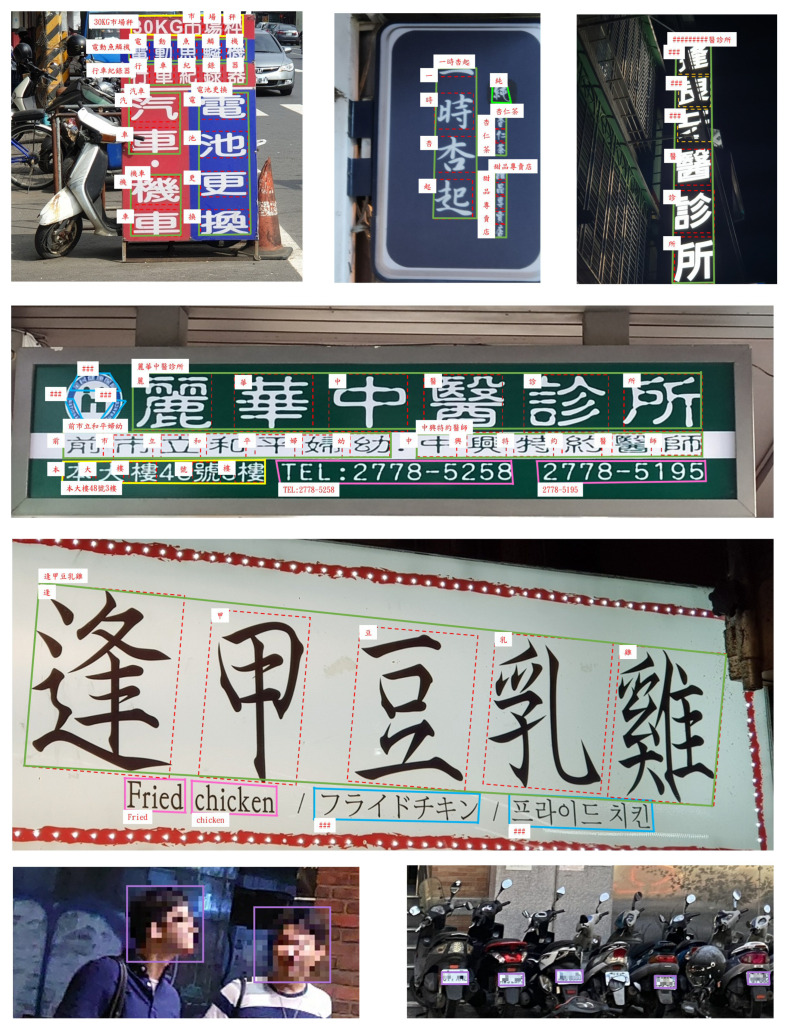
CSVT annotation examples. The polygon colors correspond to the following categories: dark green: Category 0; red: Category 1; pink: Category 2; yellow: Category 3; light green: Category 4; blue: Category 5; purple: Categories 6 and 7; orange: Category 255. The content of Category 255 shows “###” to distinguish it from the literal “#” character that may appear in text.

Typical annotation examples are shown in Figure 17. As noted previously, the dataset provides annotations for various types of text and their corresponding categories. Each word or character is annotated with a four-point polygon, recorded in clockwise order, defining its location and boundaries. This level of detail enables the precise recognition of both text regions and individual characters, which is crucial for accurate scene text detection. A total of 555,335 instances (86,070 in Category 0, 350,237 in Category 1, 41,513 in Category 2, 6320 in Category 3, 5382 in Category 4, 39,569 in Category 5, and 26,604 in Category 255) are annotated in the CSVT dataset, excluding the instances of Categories 6 and 7. CSVT contains 3938 different Chinese characters.

To account for the diverse scenarios encountered in scene text, the following annotation rules are established to ensure consistent labeling. For Chinese text, character spacing, orientation, and size are prioritized to achieve reasonable string segmentation, while fonts and colors are considered less critical. For English alphanumerics, words and numbers are grouped to preserve semantic completeness, with careful attention to spacing, particularly for numbers, and proper handling of apostrophes and hyphens. String orientation is also taken into account when determining text boundaries. Punctuation marks are treated differently for English and Chinese, reflecting the distinct conventions in their usage. Additionally, CSVT is manually annotated, which may introduce subjective judgments or occasional annotation errors. For instance, it is necessary to ensure that the four corner points of each text string or character are labeled in a clockwise order, as annotators may sometimes provide them in a counterclockwise sequence.

**Figure 18:**
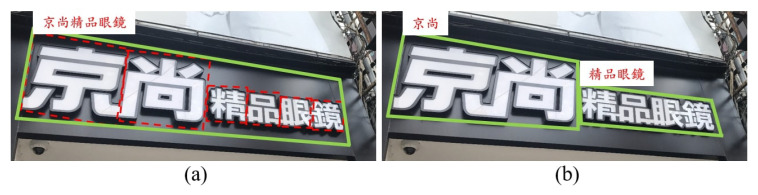
An example of string separation for text with varying sizes. The left panel (**a**) shows the original label, grouping all six characters into a single string. The right panel (**b**) shows the corrected label, where the string is divided into two segments: one with two characters and the other with four.

Labeling Chinese text presents unique challenges due to the varying sizes of characters within a string. In some cases, strings need to be split into multiple segments to ensure that characters within each segment are of similar size. A typical example is shown in Figure 18. The left panel shows a straightforward annotation, grouping all six characters into a single string. The right panel shows a more accurate annotation, where the string is divided into two segments, one containing two characters and the other four, based on the principle of maintaining similar character sizes within each segment. The dataset incorporates an automatic consistency-checking process to determine whether a string should be split. This process examines size differences among characters within a string. Specifically, characters in Category 1 are used to guide segmentation based on their bounding boxes. For each Category 0 string, all Category 1 polygons are located, and the largest and smallest characters are identified. The observed size differences provide a guideline for achieving more consistent and reasonable string segmentation.

## 5. Experimental Results

This study uses Ubuntu 18.04.5 LTS as the operating system and Python 3.7.13 as the programming language. The software stack includes PyTorch 1.10.0 with CUDA 11.1 support, Torchvision 0.11.0 with CUDA 11.1 support, and OpenCV 4.7.0. The hardware configuration consists of an Intel® (Santa Clara, CA, USA) Core^TM^ i9-9900K CPU running at 3.60 GHz and an NVIDIA® (Santa Clara, CA, USA) GeForce RTX 3090 GPU (Graphics Processing Unit) for training. CUDA 11.1 is used to enhance GPU computational performance, and cuDNN 8.0.5 is adopted to accelerate operations within the deep learning framework.

### 5.1. Training Details

Approximately 11,000 synthetic images with precise character-gap annotations, including alphanumeric content, were generated to form the training set for the initial model in the first stage. The model was trained for 100 epochs with a batch size of 3. In the second stage, weakly supervised learning incorporated real images for an additional 100 training epochs. Initial pseudo-labels were generated for these real images and iteratively refined based on the model’s confidence maps. Training alternated between synthetic and real data, with three batches of synthetic images followed by one batch of real images, enabling the model to leverage the stability of synthetic data while adapting to real-world variations. Stochastic Gradient Descent (SGD) with an initial learning rate of $2\times 10^{-4}$ was used as the optimizer, and an exponential learning rate scheduler gradually reduced the learning rate during training to promote faster convergence and mitigate overfitting.

### 5.2. Evaluation Method

#### 5.2.1. Alphanumeric Character Recognition

Most commonly used datasets provide only word-level bounding boxes and text content, lacking character-level annotations, which prevents direct evaluation of character detection accuracy. To address this, we adopt an indirect evaluation approach. For each detected character box, we calculate its overlap with all word boxes and assign it to the word box with the highest Intersection over Union (IoU), using a threshold of 0.65. A lightweight alphanumeric character recognition model based on EfficientNetV2 [38] is then applied to each detected character. The recognition results are compared with the text content of the matched word box to determine correctness.

The performance is evaluated by Recall, Precision, and F1-score defined in (5), (6), and (7) respectively. TP (True Positives) represents the number of character boxes that overlap with word boxes and have the same content. FN (False Negatives) denotes character boxes that overlap with word boxes but have different content. FP (False Positives) refers to character boxes that do not overlap with word boxes yet are incorrectly classified as overlapping. Recall considers TP representing the number of correctly identified characters, and the sum of TP and FN, reflecting the total number of characters in the dataset. Precision considers TP and the sum of TP and FP, indicating the total number of characters detected by the model. The F1-score is the harmonic mean of Recall and Precision, offering a more comprehensive evaluation of the model’s performance.

(5) $$\mathrm{Recall}=\frac{TP}{TP+FN}.$$

(6) $$\mathrm{Precision}=\frac{TP}{TP+FP}.$$

(7) $$F1-\mathrm{Score}=\frac{2\times \mathrm{Precision}\times \mathrm{Recall}}{\mathrm{Precision}+\mathrm{Recall}}.$$

#### 5.2.2. Character Count Evaluation

The validation dataset contains multilingual text, including many languages for which the recognition model has not been trained. To enable language-agnostic evaluation, we adopt a word-count-based scoring function defined as follows:

(8) $$Score=\frac{\sum_{i=1}^{N}max\left(0,1-\frac{|y_{i}-x_{i}|}{y_{i}}\right)}{N},$$

where $y_{i}$ represents the number of characters in the *i*-th word box, $x_{i}$ is the number of characters detected by the model, and *N* is the total number of word boxes. Each word box can achieve a maximum score of 1. The score for a word box decreases as the difference between $x_{i}$ and $y_{i}$ increases, with a minimum of 0. The final dataset score is computed by averaging the scores across all word boxes, providing an overall measure of character detection accuracy.

### 5.3. Validation Dataset Results

The validation datasets used in this study are primarily ICDAR2017 [31] and Total-Text [39]. ICDAR2017 comprises scene images containing text in English, Chinese, Japanese, and Korean, whereas Total-Text consists of scene images with English text in arbitrary orientations. These datasets are used to evaluate the model’s performance and to facilitate comparisons with state-of-the-art methods, including CRAFT [23], Char-Net [25], TextFuseNet [40], and CharSpotter [37].

#### 5.3.1. ICDAR2017: English and Numerical Digits

Since the ICDAR2017 dataset contains real-world scene images with text in English, Chinese, Japanese, and Korean, and evaluation protocols differ across languages, we select approximately 1500 images containing only English text and numerical digits for evaluation (Table 2). For single-character recognition, we employ our own trained model based on the EfficientNetV2 architecture [38], which is specifically designed to recognize English characters and digits. In contrast, CharSpotter [37] uses the PARSeq model [41], which is optimized for natural scene text recognition.

We adopt a single-character recognition model to evaluate character detection performance, as it provides a more direct and reliable assessment than word-level text recognition models. String recognition models, typically trained on large and diverse datasets, can exploit linguistic context to compensate for imperfect character segmentation, potentially masking detection errors. By contrast, character-level recognition relies solely on the quality of segmentation, making it a more rigorous measure of character detection effectiveness.

#### 5.3.2. ICDAR2017: Multilingual

Most existing character- and word-recognition models are primarily designed for English alphanumeric text, which presents several challenges when extending them to Chinese, Japanese, and Korean (CJK) scripts. One key reason is the relative simplicity of the English character set, which consists of only 62 classes, including uppercase letters, lowercase letters, and digits. In contrast, Chinese writing comprises thousands of characters, with approximately 4000 commonly used in daily contexts. Japanese text includes multiple writing systems, including hiragana, katakana, and kanji, while Korean is composed of 24 basic letters that combine to form hundreds of distinct characters.

In addition, CJK characters exhibit significantly more complex structural patterns than English characters, requiring substantially larger amounts of training data and fine-grained annotations to learn discriminative features for recognition adequately. This complexity greatly increases the cost and difficulty of training accurate recognition models for these languages. Given these challenges, developing precise single-character recognition models for non-English scripts remains a nontrivial task. Consequently, we adopt word count as a coarse-grained evaluation metric in our experiments, and the corresponding results are reported in Table 3.

#### 5.3.3. Total-Text

The Total-Text dataset [39] contains 300 images with multi-directional and arbitrarily shaped text. Table 4 reports the test results on this benchmark. On images with highly irregular and arbitrarily oriented text, LICS underperforms compared to CRAFT and CharSpotter, particularly when text shapes are asymmetric or structurally complex. In such cases, the current implementation of LICS is more likely to miss inter-character gaps, leading to suboptimal character segmentation and, consequently, reduced recognition accuracy.

Several factors may contribute to this performance gap, including limited diversity in training data and variations in training duration. CRAFT is trained on approximately 800,000 synthetic and real images over 50,000 training epochs, while Char-Net [25] uses a similarly sized dataset but is trained for 800 epochs. In contrast, our model is trained on approximately 11,000 synthetic images, supplemented with real images, for only 200 epochs. This substantial disparity in both data scale and training time likely affects the model’s ability to generalize to complex text layouts.

#### 5.3.4. Comparison by Word Recognition

Following common practice in scene text recognition, we further evaluate the performance of LICS using the word prediction rate, where a prediction is considered correct if the word in an image is accurately recognized. We compute this metric across multiple public datasets and compare our results with those of SAR [42], RobustScanner [43], I2C2W [27], and CharSpotter [37]. The results shown in Table 5 indicate that LICS outperforms SAR, RobustScanner, and I2C2W, but slightly lags behind CharSpotter. This difference is likely attributable to CharSpotter’s direct character detection strategy, whereas LICS infers character locations indirectly through gap detection. While effective in many scenarios, this indirect approach may introduce additional variability in complex or multi-oriented text, thereby increasing overall uncertainty in character localization.

### 5.4. Ablation Study

We conduct an ablation study on character detection using alphanumeric text from ICDAR2017. LICS starts from an initial model trained on synthetic data with character-level annotations. As shown in Table 6, models trained exclusively on synthetic datasets exhibit substantially lower detection performance. The first row (Initial model*) corresponds to an initial model trained with binary classification (background and gaps), rather than ternary classification (background, gaps, and boundary). The ternary classification strategy, originally proposed in CharSpotter [37], is designed to better separate adjacent characters. We observe that it also improves gap detection, likely because the additional boundary class facilitates the identification of small targets, i.e., the gaps. We then apply weakly supervised learning (WSL) to localize gaps. The third row (+WSL) displays the results when the detected gaps are used directly to extract characters. Performance improves significantly, demonstrating that weakly supervised learning is the most critical component of LICS. The fourth row demonstrates the feasibility of the simulated gap method described in Section 3.4. Finally, a post-processing step based on a nearest-neighbor approach is applied to group pixels into characters, which further improves accuracy.

### 5.5. Visualization of Character Segmentation Results

**Figure 19:**
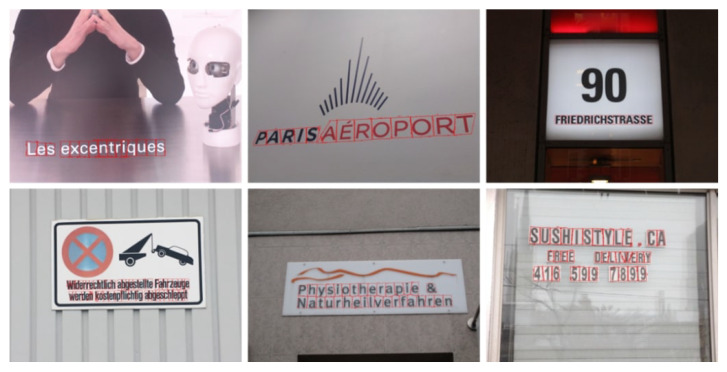
Character localization results for the English alphanumeric text of ICDAR2017.

**Figure 20:**
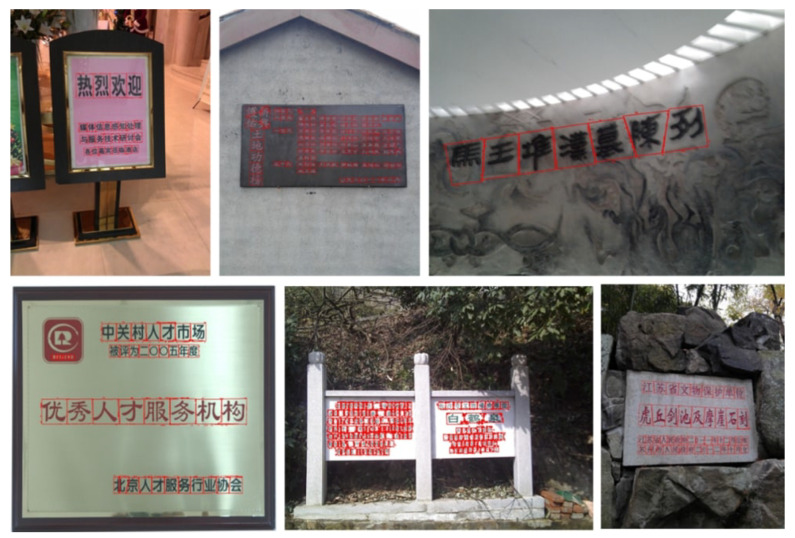
Character localization results for the Chinese text of ICDAR2017.

**Figure 21:**
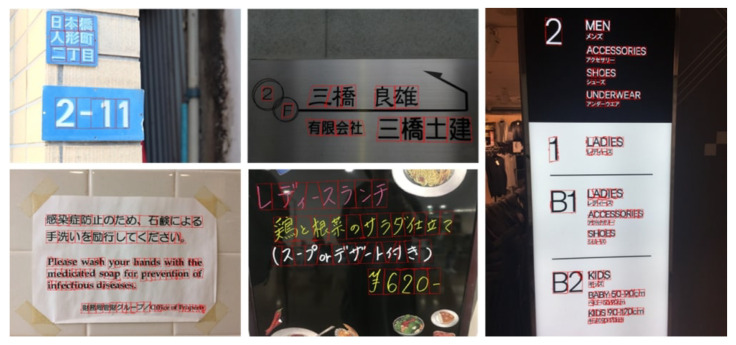
Character localization results for the Japanese text of ICDAR2017.

**Figure 22:**
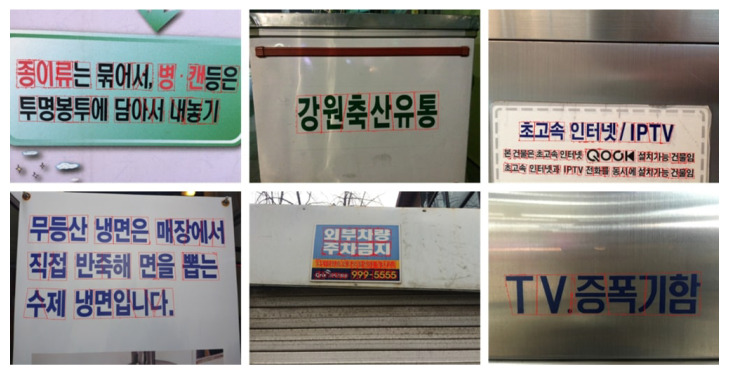
Character localization results for the Korean text of ICDAR2017.

**Figure 23:**
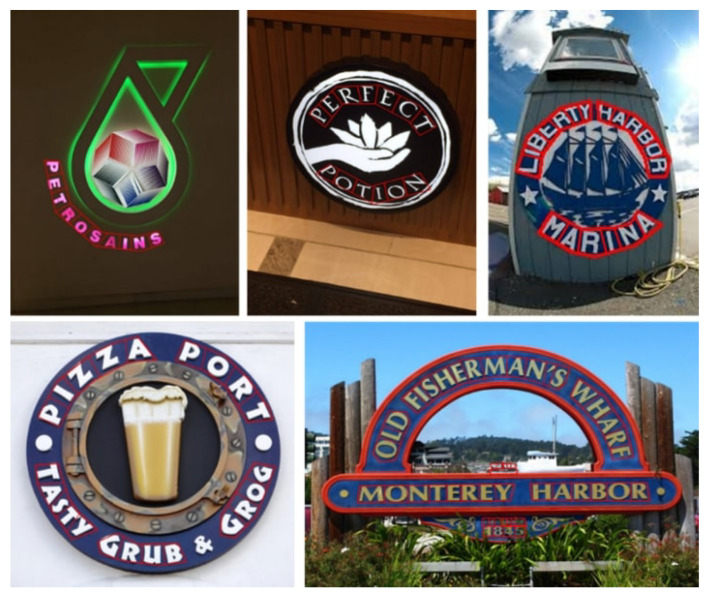
Character localization results for Total-Text.

**Figure 24:**
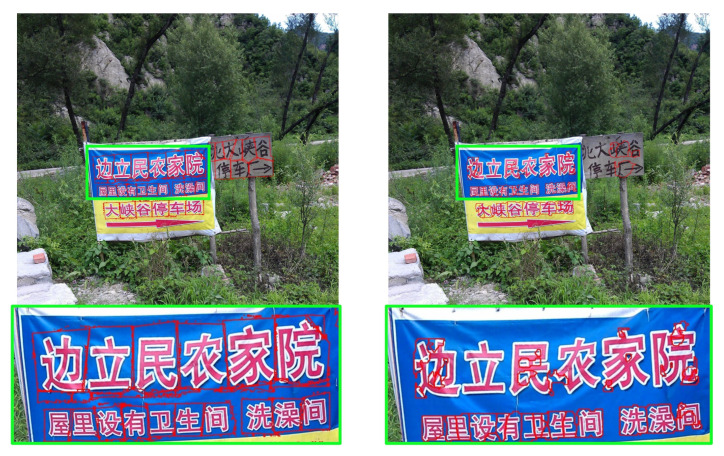
Comparison of Chinese character segmentation results of LICS (**left**) and CharSpotter [37] (**right**).

**Figure 25:**
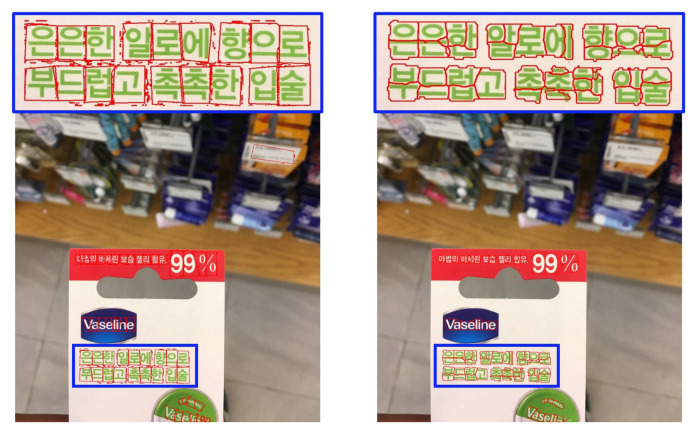
Comparison of Korean character segmentation results of LICS (**left**) and CharSpotter [37] (**right**).

**Figure 26:**
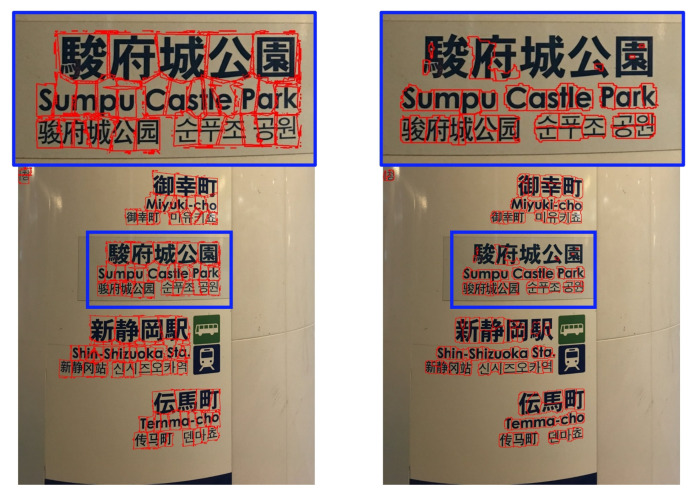
Comparison of Japanese character segmentation results of LICS (**left**) and CharSpotter [37] (**right**).

The gap detection model is visually evaluated on multilingual natural scene images. As shown in Figure 19, Figure 20, Figure 21 and Figure 22, LICS accurately segments characters in English alphanumeric text, as well as Chinese, Japanese, and Korean text. The model also performs well on neatly handwritten text (Figure 21) and multi-oriented scene text (Figure 23). Compared with CharSpotter, LICS demonstrates superior character localization across multilingual scripts, as illustrated in Figure 24, Figure 25 and Figure 26. Despite substantial variations in character shapes across languages, the consistent presence of inter-character gaps enables LICS to generalize effectively.

These results indicate that LICS not only achieves accurate character segmentation in English alphanumeric text but also reliably localizes individual characters in natural scene images containing diverse scripts, highlighting its strong generalization capability. In addition, the model exhibits robust performance on neatly handwritten text and text with arbitrary orientations. Overall, the proposed gap detection model delivers high-quality character segmentation in multilingual text images, producing bounding boxes that closely align with true character positions. This provides clean and reliable input for subsequent character recognition stages, thereby reducing both computational complexity and error propagation in downstream recognition tasks.

**Figure 27:**
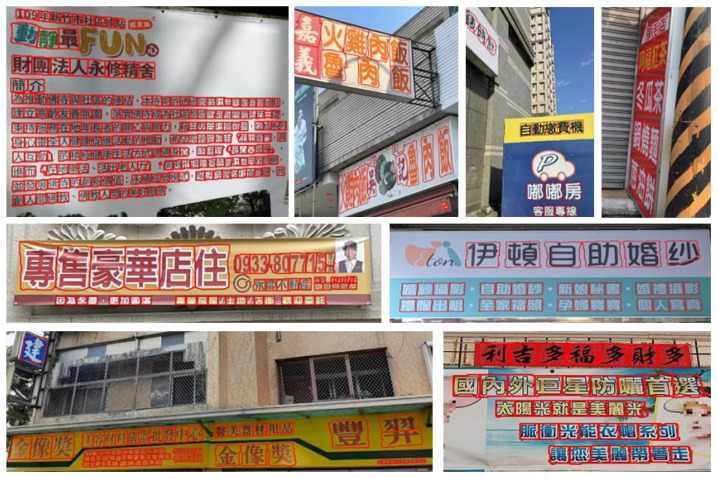
The character localization results of CSVT.

The character localization results on CSVT are demonstrated in Figure 27, from which we can see that most characters can be retrieved successfully. Then, a lightweight character recognition model can be employed to analyze the scene text and facilitate related applications.

**Figure 28:**
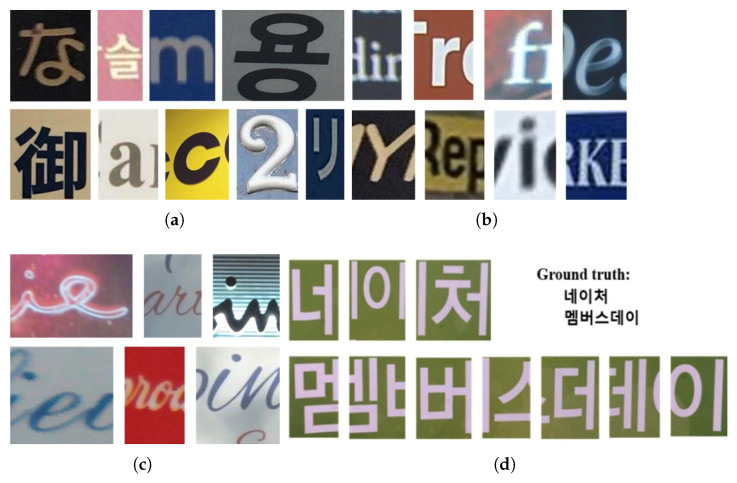
Examples of segmented characters; (**a**) normal cases; (**b**) dense scene text; (**c**) connected handwritten text; (**d**) incorrect segmentation of Korean characters.

By detecting inter-character gaps and computing their centers to approximate character centers, followed by character segmentation using a nearest-neighbor algorithm, our approach generally achieves satisfactory segmentation results across multiple languages in natural scene images, as shown in Figure 28a. Nevertheless, several limitations remain. First, small characters that are closely spaced may lead to missed gap detections, as illustrated in Figure 28b. In such cases, a segmented character may incorrectly include portions of adjacent characters, reducing the reliability of subsequent character recognition. Similar issues are observed for handwritten and cursive text, as shown in Figure 28c. Training the model on a large-scale dataset of authentic handwritten text could help alleviate this limitation. Our experiments further reveal that Korean text is more prone to segmentation errors than other languages. As illustrated in Figure 28d, this issue likely stems from the structural properties of Korean characters, which are composed of multiple constituent letters arranged with internal spacing. The model may mistakenly interpret these internal spaces as inter-character gaps, leading to incorrect segmentation.

**Figure 29:**
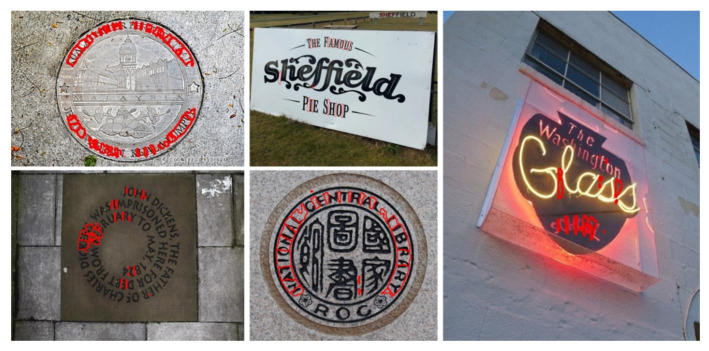
Complex multi-oriented text may result in poor detections of inter-character gaps.

When processing multi-oriented text in scene images, several factors affect the accuracy of gap detection. Densely packed text or text with irregular and asymmetric structures can significantly degrade detection performance, as illustrated in Figure 29. Furthermore, scenes containing text at multiple orientations and with complex shapes present additional challenges, since inter-character gaps in such cases often deviate from simple linear patterns and instead exhibit highly diverse spatial configurations. These challenges highlight the need to enhance the model’s feature extraction capabilities to better capture subtle variations and structural irregularities in character gaps. Moreover, increasing the diversity of training data is essential to improve the model’s generalization ability and robustness across a wide range of complex text layouts. Despite these limitations, which should be addressed in future work, LICS demonstrates that character-level processing can effectively reduce reliance on explicit orientation modeling. In contrast, string-level processing typically requires accurate orientation estimation in advance and the training of separate models for horizontal and vertical text.

## 6. Conclusions and Future Work

In this paper, we presented LICS (Locating Inter-Character Spaces), a novel approach to multilingual scene text detection that exploits inter-character gaps as universal structural cues. Unlike conventional methods that rely heavily on language-specific character shapes or word structures, LICS leverages the more consistent geometric patterns of spaces between characters, resulting in a language-agnostic framework with improved generalizability across scripts. We introduced a two-stage training strategy: in the first stage, the model is trained on synthetic datasets with accurate inter-character gap annotations; in the second stage, weakly supervised learning is applied to real-world datasets containing only word-level annotations. This design substantially reduces annotation costs while enabling effective gap detection without requiring character-level annotations in target languages. Extensive experiments on multiple multilingual benchmarks demonstrate the effectiveness of the proposed method. LICS consistently outperforms traditional text detection approaches, particularly in challenging multilingual settings that involve diverse scripts, such as Chinese, Japanese, and Korean. The results further demonstrate that LICS strikes a favorable balance between precision and recall, underscoring its potential for real-world scene text detection applications.

Despite its strong performance, several limitations remain. In particular, datasets such as Total-Text pose challenges due to their highly irregular text orientations and curved layouts, which are not yet handled optimally by the current model. Future work could focus on improving gap detection in such scenarios by incorporating richer geometric representations or more diverse training data to incorporate curvature-aware gap modeling. Additionally, although the weakly supervised framework effectively reduces annotation requirements, the quality of pseudo-labels remains dependent on the initial model’s accuracy. Future research may explore more robust pseudo-label generation strategies, such as improved confidence estimation or active learning mechanisms. Furthermore, extending the training data to include a broader range of languages and scripts, such as Arabic, Hindi, and Thai, would enhance the model’s robustness and cultural coverage. Finally, integrating LICS with downstream tasks such as text recognition and scene understanding would enable a more comprehensive scene text analysis pipeline, with promising applications in augmented reality, autonomous driving, and real-time translation systems.

## Figures and Tables

**Table 1:** Language Categories.

Category	String Language	Example
0	Chinese string	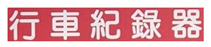
1	Chinese character	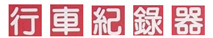
2	English alphanumerics	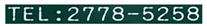
3	Mixed string	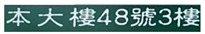
4	Single character (Exist separately)	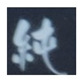
5	1. Other languages (such as Japanese, Korean, etc.) 2. Blurred single-line string	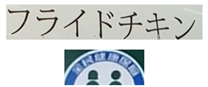
6	License plate	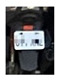
7	Face	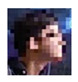
255	Don’t-Care	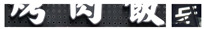

**Table 2:** Detection results for the alphanumeric dataset of ICDAR2017.

Method	Recall	Precision	F-Score
CRAFT [23]	65.11%	74.03%	69.28%
Char-Net [25]	73.95%	79.41%	76.58%
TextFuseNet [40]	43.51%	83.28%	57.15%
CharSpotter [37]	81.04%	86.03%	83.46%
LICS	**82.79%**	**87.42%**	**85.04%**

Bold font indicates the best result.

**Table 3:** Evaluation of detection results for multilingual text in ICDAR2017.

Method	English	Chinese	Korean	Japanese
CRAFT [23]	83.47%	60.95%	52.66%	57.70%
Char-Net [25]	86.03%	71.49%	63.09%	64.58%
TextFuseNet [40]	70.44%	50.44%	43.78%	44.30%
CharSpotter [37]	**88.63%**	68.46%	68.88%	62.29%
LICS	88.01%	**81.65%**	**76.56%**	**85.09%**

Bold font indicates the best result.

**Table 4:** Detection results on Total-Text.

Method	Recall	Precision	F-Score
CRAFT [23]	80.29%	82.17%	81.21%
Char-Net [25]	73.57%	81.10%	77.15%
TextFuseNet [40]	59.35%	**84.63%**	69.77%
CharSpotter [37]	**83.04%**	82.17%	**82.60%**
LICS	78.06%	83.71%	80.02%

Bold font indicates the best result.

**Table 5:** Comparison with recent scene text recognition research.

Method	Recall
SAR [42]	78.40%
RobustScanner [43]	77.82%
I2C2W [27]	81.53%
CharSpotter [37]	**84.28%**
LICS	82.11%

Bold font indicates the best result.

**Table 6:** Ablation Study on multilingual text in ICDAR2017.

Method	Recall	Precision	F-Score
Initial model *	53.67%	73.23%	61.94%
Initial model	54.71%	76.61%	63.83%
+WSL	78.58%	82.73%	80.60%
+Simulated gap	80.73%	85.25%	82.93%
+Post-processing	82.79%	87.42%	85.04%

* indicates that the model is trained using two classes: background and gap.

## Data Availability

Restrictions apply to the availability of these data. icdar2017 [31]: https://www.kaggle.com/datasets/dh2571/icdar17 (accessed on 10 May 2025); Total-Text [39]: https://github.com/cs-chan/Total-Text-Dataset (accessed on on 10 May 2025); CSVT: https://github.com/ncumsplab/CSVT (accessed on on 14 November 2025).

## Abbreviations

The following abbreviations are used in this manuscript:

LICS	Locating Inter-Character Spaces
ICDAR	International Conference on Document Analysis and Recognition
IoU	Intersection over Union
JSON	JavaScript Object Notation
UTF-8	8-bit Unicode Transformation Format
CRAFT	Character Region Awareness for Text detection
CSVT	Character-Labeled Street View Text
